# Assessments of functional outcomes and its determinants among bipolar disorder patients in Northwest Ethiopia comprehensive specialized hospitals: a multicenter hospital-based study

**DOI:** 10.1186/s12991-023-00444-3

**Published:** 2023-04-06

**Authors:** Melak Erara Mengistu, Simegnew Handebo Berassa, Abebe Tarekegn Kassaw, Ephrem Mebratu Dagnew, Gizework Alemnew Mekonen, Mequanent Kassa Birarra

**Affiliations:** 1 Department of Clinical Pharmacy, School of Pharmacy, University of Gondar-College of Medicine and Health Science, Gondar, Ethiopia; 2 School of Public Health, St. Paul’s Hospital Millennium Medical College, Addis Ababa, Ethiopia, Addis Ababa, Ethiopia; 3 Department of Pharmacy, College of Medicine and Health Science, Woldia University, P.O. Box: 400, Woldia, Ethiopia; 4 Department of Pharmacy, College of Medicine and Health Science, Debre Markos University Debre Markos, Debre Markos, Ethiopia

**Keywords:** Functional outcomes, Impairment, Bipolar disorder, Northwest Ethiopia

## Abstract

**Introduction:**

Bipolar disorder is a severe and chronic mental illness that could continue for a lifetime. Although it is a leading cause of disability and impairments for significant numbers of patients, the levels of functional outcomes have not been studied in Ethiopia. Therefore, this study aimed to assess the functional outcome levels and associated factors among bipolar disorder patients in Northwest Ethiopia.

**Method:**

Hospital-based cross-sectional study was employed among bipolar disorder patients attending psychiatric clinics, in Northwest Ethiopia, from April to June 2021. Systematic random sampling was used to get respondents. Descriptive and inferential statistics were done. Data were entered into EpiData version 4.6.02 and exported to SPSS Version 22 for analysis. Bivariable and multivariable binary logistic regression analysis was used to identify the factors associated with functional outcome levels, and *p* value < 0.05 was considered significant with 95% CI.

**Result:**

Of the total 423 study participants approached, only 411 completed the questionnaire, with a response rate of 97.2%. The median (IQR) level of functional outcome was 6 (0–22) and 40% of the study subjects were impaired. Leisure time was the most normal functioning domain (92.2%), whereas cognitive (43.5%) and occupational (41.6%) domains were the most impaired domains. Unemployment (AOR (95%CI) = 3.9 (1.46–10.49), obesity (AOR (95% CI) = 6.5 (1.22–34.58), depressed and manic mood phases (AOR (95%CI) = 5.2 (2.84–9.35) and (AOR (95%CI) = 7.8 (3.31–18.34) respectively, medication non-adherence (AOR (95% CI) = 3.2 (1.71–6.05), and relapsed once or ≥ twice (AOR (95%CI) = 2.2 (1.25–3.98) and (AOR (95%CI) = 8.3 (2.73–25.30), respectively, were some of the important predictor variables that were significantly associated to the functional impairments levels.

**Conclusion:**

The median of functional outcomes levels was found in an acceptable range; however, significant numbers of bipolar patients were functionally impaired. Moreover, patients still need unrestricted interventions in the cognitive and occupational functional domains. Socio-demographic, clinical, medication, and psychosocial variables were significantly associated with functional outcomes. Bipolar patients need to be followed and managed to improve their functional outcome and all stakeholders should be involved to achieve the recommended levels.

**Supplementary Information:**

The online version contains supplementary material available at 10.1186/s12991-023-00444-3.

## Introduction

Bipolar disorder (BD) is a chronic and recurrent illness that can lead to severe disruptions in family, social, and occupational functioning [1]. Numerous hypotheses have been proposed to explain the underlying patho-etiology of BD, but the mechanisms of sub-serving disease onset and progression remain largely unknown. More recently, immune dysfunction has been implicated in the patho-etiology of BD [2]. Bipolar disorder has the highest rate of suicide among all mental health conditions. This rate is about 10–30 times higher in people living with bipolar disorder than in the general population [3]. Evidence obtained from randomized controlled trials and systematic reviews showed that low-dose buprenorphine is an effective, well-tolerated medication in reducing serious suicidal ideation [4], even though the suicidal ideation in patients with bipolar disorder is treated by buprenorphine the functional impairment is still a major problem.

In patients with BD, functional impairment is an important driver of disability and can remain even when symptomatic reduction has been accomplished [5]. Mental health services have been shifted from an emphasis on treatment focused on reducing symptoms to an approach that takes into account both well-being and functioning [6]. In the former report, bipolar disorder has better functional consequences other than mental illnesses, since presumed lack of cognitive impairment and normal functioning between episodes [7], thus makes functional outcome has been given little attention in patients with bipolar disorder than in other mental illness. But, in contrast to earlier studies, currently, a significant degree of functional dysfunction had been pointed out even when patients are normal in mood status [8].

Being functional means having the ability to work, study, live independently, participate in romantic and recreational activities [9], and also be able to return to the degree of functioning that was present before the most recent episodes [8]. The WHO International Classification of Functioning, Disability, and Health states that functioning and disability are multidimensional concepts that involve the capacity to manage physical functions, carry out self-care tasks, engage in everyday activities, and manage environmental factors that either facilitate or impede these experiences [10]. Patients with bipolar disorder (BD) may continue to be disabled as a result of lack of treatment, which could potentially impair their functioning and productivity as well as reduce their overall quality of life.

The prevalence of moderate or severe disability for BD is projected to be 22 million people worldwide, according to the WHO report, making it the 12th most common cause of disability overall [11]. Patients with BD report a range of issues with their ability to function at work [12], along with substantial work impairment during a sizable amount of their long-term illness course [13], and high unemployment rates [14]. They are less active socially [15], have strained family ties [16], and are more likely to be widowed, separated, or divorced [17]. Caregivers who express significant hardship and distress related to relationships and daily tasks are also affected negatively by BD [18]. As a result, functional outcome is defined as the capacity to maintain interpersonal connections and fulfill role expectations in all domains of life and is more significant than the syndromal outcome for many patients with BD and their families [19].

Although BD-associated functional impairment has been extensively studied in developed countries, data were scarce in developing nations, especially in Africa, Ethiopia. A study was done in southwestern Ethiopia, Butajira, and it was a community survey and did not include potential factors, like the level of treatment adherence, and sociocultural factors like social support. Furthermore, the study was done on specific age groups and rural areas and was not fully reflect the course of illness in major cities. So we addressed the above limitations of the previous study. Therefore, this study aimed to determine the level of functional outcomes and determinants among adult bipolar disorder patients. The finding of this study will support and guide clinical decisions in the management of bipolar disorder management policies and also provide a baseline for further similar studies and, furthermore, will inform many stakeholders about the potential gaps and barriers to functional outcomes and determinants of the outcome among bipolar disorder patients.

## Materials and methods

### Study design, study period, and setting

The hospital-based multicenter cross-sectional study was conducted over three consecutive months from April to June 2021. The study participants were enrolled from three Comprehensive specialized hospitals in Northwest Ethiopia namely, the University of Gondar Comprehensive Specialized Hospital (UoGCSH), Felegehiwot Comprehensive Specialized Hospital (FHCSH), and Tibebegion Comprehensive Specialized Hospital (TGCSH).

The UoGCSH is one of the oldest Ethiopian academic institutions. It has more than 1000 beds and provides its service in a range of specialties for more than 5 million people living in Gondar town and neighboring Woreda and zones. The annual follow-up of bipolar patients in the outpatient psychiatry clinic was 850 patients. FHCSH and TGCSH are found in Bahir Dar city, the hospitals give services to the residents of Bahir Dar and nearby zones, which have a collective population of about 11 million people [20]. Annually, 780 and 320 bipolar patients had outpatient follow-ups visit at the psychiatric units of FHCSH and TGCSH, respectively.

### Study population and sampling

This study included patients with bipolar illness who were 18 years of age or older, who visited the psychiatric unit of the chosen hospitals for follow-up, must have passed the insight criteria, and received therapy for at least one month at the selected hospitals. The insight of the study participants was assessed using the insight assessment tool [21], in so doing patients can be classified as having the insight or no insight. Exclusion criteria for study participants who are not willing to give consent are acutely ill, inability to communicate, having a physical disability, having insufficient medical records, having dementia or other forms of cognitive impairment, and having difficulties obtaining consent. The single population formula was used to determine the sample size for this investigation. Because there is not any published study about the prevalence of functional outcomes of bipolar illness patients in the study area, 50% of the prevalence was used as a proportion (*P*). About 384 participants were calculated using the marginal error of 5% (*W* = 0.05) at the 95% confidence interval (*Z*/2 = 1.96); however, allowing for potential non-response and a 10% contingency, ultimately the sample size of 423 was included in the study. The study subjects were chosen using a systematic random sampling method.

Patients with bipolar disorder are recommended to see the psychiatry clinic for a minimum of everyone month and a maximum of every three months. During the three months, 850 patients visited UoGCSH, 780 at FHCSH, and 320 at TGCSH, and a total of 1950 patients during three months at the three institutions. Finally, 184, 169, and 70 patients from UoGCSH, FHCSH, and TGCSH, respectively, were approached to participate in the study using proportional allocation. Among 423 participants approached, only 411 subjects fulfilled the inclusion criteria, but seven subjects have withdrawn from the interview due to different reasons and five subjects had no insight. Given that the sample was taken within three months, the sampling fraction (*k* interval) is 1950/423 = 5 (Approximate). As a result, the initial study subject was picked by lottery, and subsequent study participants were chosen from every fifth person, whose associated medical records were subsequently gathered, and from which pertinent data were retrieved. The medical records of the study respondents who met the inclusion criteria were also reviewed, and if one medical record was found to be ineligible, the next was chosen. The same method was used throughout the whole data-gathering process. Each selected responder was also interviewed.

### Definitions of terms

#### Functional outcome

Patients who regained their overall and specific function equivalent to those who had before their illness.

#### Functionally no impaired

The capacity to work, study, live independently, and engage in recreational activities and interpersonal relationships, a FAST total score of 0–11.

#### Functionally impaired

A difficulty in functioning in one or more life domains, as experienced by an individual with a health condition in interaction with contextual factors, a FAST total score of > 11.

#### Social support

Having physical and emotional support given by family, friends, co-workers, and other people in times of need or crisis.

#### Current substance use

Use of alcohol, chat, tobacco, and others for non-medical use in the last 3 months.

#### Lifetime substance use

Use of alcohol, chat, tobacco, and others for non-medical use in the lifetime.

#### Suicidal ideation

Individuals who were seriously thinking about committing suicide within the last month.

#### Suicidal attempt

Participants who attempted to commit suicide within the last month.

#### Insight

A capacity to recognize and accept that they are suffering from a mental illness.

### Data collection instruments and procedures

After reviewing a variety of similar literature, the data collection tool for this study was initially created in English. The translation was then made into Amharic, which was then back translated into English to assure uniformity. The instrument was divided into five sections: (I) an insight assessment tool; (II) socio-demographic information of study participants; (III) clinical and psychosocial characteristics of patients; (lV) adherence levels; and (V) instruments to measure functional outcomes for patients. The training was provided to data collectors about the purpose of the study, the procedure and tool used for collecting data, and ethical concerns. Participants' medical records and interviews were examined to gather pertinent information. However, if one of the accessible medical records was ineligible, the next one was taken into consideration. This strategy was used throughout the time of data collection. The functional assessment short test (FAST) tool was used to evaluate the functional outcome. It was developed for the evaluation of the main difficulties presented by psychiatric patients, particularly those with bipolar illness. The Medication Adherence Rating Scale (MRSA) tool was used to evaluate the adherence of patients to their prescribed medications. The MARS is a 10-item self-reporting multidimensional instrument describing three dimensions: medication adherence behavior (items 1–4), attitude toward taking medication (items 5–8), and negative side effects and attitudes to psychotropic medication (items 9–10). A different questionnaire was used to gather data on the factors that influence functional outcomes.

### Measurements of functional outcomes

The FAST tool was used to measure the functional outcome of bipolar patients. Unlike the other instruments, functioning has focused on global measures of functional recovery, this tool can measure specific domains of psychosocial functioning. FAST is a brief instrument designed to assess the main functioning problems experienced by bipolar patients. It comprises 24 items that assess impairment or disability in six specific areas of functioning: autonomy, occupational functioning, cognitive functioning, financial issues, interpersonal relationships, and leisure time.

All of the items are rated using a 4-point scale, 0 = no difficulty, 1 = mild difficulty, 2 = moderate difficulty, and 3 = severe difficulty. The global score is obtained when the scores of each item are added up. The higher the score, the more serious the difficulties are.

The following cutoff points for both overall and specific domains were calculated: autonomy (cutoff > 1), occupational functioning (cutoff > 1), cognitive functioning (cutoff > 2), financial issues (cutoff > 1), interpersonal relationships (cutoff > 3), and leisure time (cutoff > 3); the overall FAST cutoff points is > 11; individuals with a score above this threshold are considered to have functional impairments. The FAST score varied from 0 to 72, with 72 denoting the least functional outcome and 0 denoting normal functioning. A threshold score of 11 indicates the presence of a considerable disability, and a higher score indicates a greater disability. In addition, the FAST score also had cutoff points to determine the degree of severity of functional outcomes. Scored 0–11 for no impairment, 12–20 for mild impairment, 21–40 for moderate impairment, and 41–72 for severe impairment [22].

### Data quality management

After searching related literature, data collection tools were developed. The tools were modified to take into account regional clinical situations. Three language specialists reviewed the questionnaire's face validity for the questions' clarity. After that, 5% of study participants were pretested for the study's content, design, dependability, and comprehension; these individuals were excluded from the final analysis. Utilizing the WHO's recommendations, sociocultural adaptation was carried out, and adjustments were made in response to feedback. As a result, while still giving accurate data, the study was simple to comprehend and respond to. At each stage of the data-gathering process, the data collectors and supervisors checked the quality of the data. The 24 items in the FAST tools were examined for internal consistency, and they demonstrated strong consistency with a Cronbach's alpha of 0.963.

### Data entry and analysis

The data were examined for accuracy and completeness before being coded, inputted, and exported to EpiData version 4.6.02 for analysis. The characteristics of the study participants were described using descriptive statistics, means, proportions, tables, and figures, which were also utilized to convey the study findings. Before entering variables into logistics regression, the Chi-square test was done to ensure that they met the Chi-square assumption. Then, variables from the basic logistics regression that had a *p*-value of less than 0.2 were included in the multivariable logistics regression. An adjusted odds ratio (AOR) and 95% confidence interval (CI) were used to evaluate the strength and direction of connection between the dependent and independent variables to discover factors related to functional impairments. A *p*-value of 0.05 or blew was used to indicate statistical significance. The Hosmer and Lemeshow test of goodness of fit was used to determine whether the models were adequate.

### Ethical consideration

Ethical approval was obtained from the Institutional Review Board (IRB) of the University of Gondar with a reference number of SOP/257/2021. Then, a letter from the clinical director of each hospital was also received before beginning the work in their facilities. The purpose of this study was explained to the study participants and informed consent was obtained. Both verbal and written form were given by participants to indicate their willingness to participate. Participants who were unwilling to participate in the study and those who wanted to drop out at any point during the interview were informed that they could do so without any restriction. Confidentiality regarding patients was assured in such a way that the data were used for the study purpose only. Any identifiers of the study participants were not recorded. All procedures were followed according to the ethical principle of the Declaration of Helsinki rules and regulations.

## Results

### Socio-demographic characteristics of participants

Of the total 423 study participants approached, only 411 completed the questionnaire, with a response rate of 97.2%. More than half of the subjects were females (55.7%). The mean (± SD) age of the participants was 33.7 ± 11.5 years and nearly below half (46.5%) were married. One-fourth (25.3%) of the participants were housewife and (29.4%) had completed their primary education (1–8). Among the participants, more than one-half (57.4%) were urban dwellers and the majority (93%) were living with their families; however, 14.8% were homeless **(**Table 1**).**

**Table 1:** Socio-demographic characteristics of bipolar disorders participants in Northwest Ethiopia, 2021 (*N* = 411)

Variables	Frequency(%)
Sex	
Male	182 (44.3)
Female	229 (55.7)
Age	
18–24	94 (22.9)
25–34	161 (39.2)
35–44	77 (18.7)
45–54	50 (12.2)
≥ 55	29 (7.1)
Study area	
Gondar	180 (43.8)
Felegehiwot	165 (40.1)
Tibebegion	66 (16.1)
Residence	
Urban	236 (57.4)
Rural	175 (42.6)
Religion	
Orthodox	350 (85.2)
Muslim	54 (13.1)
Others	7 (1.7)
Marital status	
Single	169 (41.1)
Married	191 (46.5)
Widowed	11 (2.7)
Divorced	40 (9.7)
Education	
Illiterate	106 (25.8)
Primary (1–8)	121 (29.4)
Secondary (9–12)	83 (20.2)
College and above	101 (24.6)
Occupation	
Civil servant	60 (14.6)
Private worker	24 (5.8)
Farmer	63 (15.3)
Housewife	104 (25.3)
Unemployed	87 (21.2)
Student	73 (17.8)
No of the family	
≤ 5	304 (74)
> 5	107 (26)
Living with	
With family	382 (93)
Alone	29 (7)
Shelter	
Yes	350 (85.2)
No	61 (14.8)

### Clinical and psychosocial characteristics of study participants

The majority of the study subjects (76%) were bipolar I disorder (BID). Nearly one-half (52.6%) of the participants were in the euthymic phase and more than one-third (37.2%) were in the manic phase. About three-fourths (76.4%) were within their healthy weight and only 20.4% were with other medical comorbidities. The mean (± SD) age of illness onset was (28.1 ± 10.2) years and nearly half (45.3%) of them stayed with the disorder for the mean (± SD) of 2.33 ± 0.91 years. The mean duration of treatment was (± SD) 2.1 ± 0.86 years. Thirty-seven percent of the participants experienced at least one relapse per year and (37.2%) were also hospitalized at least once in their life due to this disorder. The majority of the respondents (62.3%) had a moderate level of social support. 18% of the study participants were thought to have suicide and 6.1% were suicide attempts. One-half of the participants (51%) were lifetime substance users (Table 2**).**

**Table 2:** Clinical and psychosocial characteristics of bipolar disorders patients in Northwest Ethiopia, 2021 (*N* = 411)

Variables	Frequency (%)
Type of BD	
BD-I	312 (76)
BD-I plus psychotic feature	80 (19.5)
BD-II	19 (4.6)
Current mood status	
Euthymic phase	216 (52.6)
Manic phase	153 (37.2)
Depressive phase	42 (10.2)
BMI	
Normal	314 (76.4)
Underweight	49 (12)
Overweight	40 (9.7)
Obese	8 (1.9)
Comorbidity	
Yes	84 (20.4)
No	327 (79.6)
Age of onset	
≤ 19	71 (17.3)
20–30	221 (53.8)
31–40	72 (17.5)
41–50	35 (8.5)
> 50	12 (2.9)
Disease duration	
≤ 1 year	71 (17.3)
1–5 year	186 (45.3)
5–10 year	103 (25.1)
> 10 year	51 (12.4)
Treatment duration	
≤ 1 year	104 (25.3)
1–5 year	193 (47)
5–10 year	84 (20.4)
> 10 year	30 (7.3)
No. of relapse since diagnosis	
No relapse	132 (32.1)
One relapse	86 (20.9)
Two relapses	80 (19.5)
≥ 3 relapse	113 (27.5)
No of relapse per year	
No relapse	234 (56.9)
One relapse	153 (37.2)
≥ 2 relapse	24 (5.8)
No of hospitalization	
No	184 (44.8)
Once	153 (37.2)
Twice	57 (13.9)
≥ Three	17 (4.1)
Social support	
Poor	146 (35.5)
Moderate	256 (62.3)
Strong	9 (2.2)
Family history of BD	
Yes	50 (12.2)
No	361 (87.8)
Suicide ideation	
Yes	74 (18)
No	337 (82)
Suicide attempt	
Yes	25 (6.1)
No	386 (93.9)
Lifetime substance use	
Yes	210 (51)
No	201 (49)
Current substance use	
Yes	51 (12.4)
No	360 (87.6)

### Medication characteristics of the study participants

**Fig. 1:**
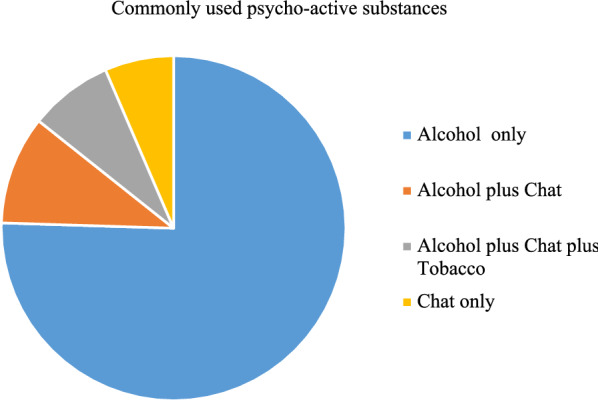
Magnitude of psycho-active substance use among bipolar disorder patients Northwest Ethiopia, 2021 (*N* = 411)

In the current study, more than two-thirds of the respondents (68.1%) were on a mood stabilizer (MS) combined with antipsychotics (AP) and 15.3% were on AP alone. Among the combinations of medications a combination of sodium valproate and risperidone (34.3%) was the most commonly prescribed medication followed by sodium valproate and haloperidol (7.6%). On the other hand, risperidone was the most commonly prescribed drug as a monotherapy regimen (6.6%). Among the study participants, more than half (51.1%) accessed their medications through payment, and (44.8%) had a history of medication discontinuation. More than half (51.8%) of the study subjects had taken traditional remedies, and holly water was the most common among traditional remedies (47%). The most frequently used psycho-active substance in their lifetime was alcohol (38.5%) (Fig. 1**).**

In terms of adherence, more than three fourth (77.9%) of participants have adhered to their medication. The mean ± SD adherence level was 6.71 ± 1.92 out of 10 scores, and most of them scored the cutoff points six and above from the ten measurements. A Spearman was tested to determine the associations between the functional impairment levels of BD patients and their medication adherence levels. The test indicated that there was a moderate, negative correlation between the two variables (*r*_s_ = − 0.384; *p* < 0.001) (Table 3).

**Table 3:** Medication characteristics of bipolar patients in Northwest Ethiopia, 2021 (*N* = 411)

Variables	Frequency (%)
Current regimen types	
MS plus AP	280 (68.1)
AP alone	63 (15.3)
MS alone	25 (6.1)
MS plus AP plus AD	22 (5.4)
MS plus AD	12 (2.9)
AP plus AD	9 (2.2)
No of previous medications	
One	99 (24.6)
Two	262 (63.1)
≥ three	50 (12.3)
No of current medications	
One	89 (21.7)
Two	310 (75.4)
≥ Three	12 (2.9)
Adherence	
Non-adherent	91 (22.1)
Adherent	320 (77.9)
Medication acquisition	
Health insurance	201 (48.9)
Out of pocket	210 (51.1)
Medication discontinuation	
Yes	184 (44.8)
No	227 (55.2)
Traditional medicine	
Yes	213 (51.8)
No	198 (48.2)

*AP* antipsychotics, *AD* anti-depressant, *MS* mood stabilizer

### Prevalence of functional impairment levels

**Fig. 2:**
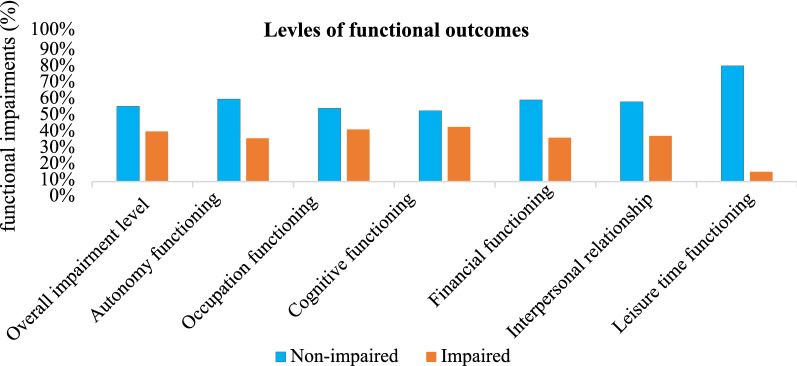
Functional impairments of BD patients in Northwest Ethiopia, 2021 (*N* = 411)

The overall median (IQR) score of functional levels was 6 (0–22) and 40% of the study patients were functionally impaired with 95%CI of (34.7–44.2). Among the specific domains, leisure time was the least impaired domain (7.8%), followed by autonomy (34.5%) and financial (35%). In contrast, the most compromised domain was cognitive (43.5%) followed by occupational (41.6%) functioning (Fig. 2). Based on the FAST cutoff points, 60% of the samples with no impairment scored 0–11, 12.3% with mild impairment scored 12–20, 19.1% with moderate impairment scored 21–40, and 8.5% with severe impairment scored 41–72.

### Factors associated with functional impairment

In the multivariate logistics regression analysis the presence of a significant association between functional outcome levels and seven independent variables, namely marital status, occupation, study area, BMI, current mood status, relapse per year, suicidal ideation, current substance use, medication adherence, and medication acquisition, were found. The functional impairment levels of married individuals were 62% lower compared with their single respondents (AOR (95%CI) = 0.38 (0.17–0.86). Similarly, the odds of the impairment status for unemployed subjects were about 2.9 times higher than civil servants (AOR (95%CI) = 3.91 (1.46–10.49). Regarding the sites where the samples had drawn, respondents from Felegehiwot and Tibebegion had 1.8 and 2.4 times higher risks of getting impaired compared with individuals from Gondar (AOR (95%CI) = 2.82 (1.49–5.33) and (AOR (95%CI) = 3.38 (1.59–7.18), respectively. In addition, obese individuals had an increased risk of having impaired functioning about 5.5 times higher than subjects with normal weight, with (AOR (95%CI) = 6.49 (1.22–34.58). In terms of mood status, the odds of impairment levels were 4.2 and 6.8 higher among individuals with manic and depressive phases compared with individuals with euthymic phase, (AOR (95%CI) = 5.15 (2.84–9.35) and (AOR (95%CI) = 7.79 (3.31–18.34). The odds of the bipolar patient's impairment levels whose symptoms were relapsed once and relapsed twice and more had a risk of impairment outcomes 2.2 and 8.3 times compared with patients with no relapsed symptom ever, with (AOR (95%CI) = 2.24 (1.25–3.98), and (AOR (95%CI) = 8.30 (2.73–25.30), respectively. On the other hand, the odds of functional impairment were 58% lower in individuals who had no suicidal ideation history compared with subjects who had those thoughts of suicide (AOR (95%CI) = 0.42 (0.216–0.795). Similarly, the odds of impairment status were lowered by 59% in patients with no history of recent substance use than the history of substance users with (AOR (95%CI) = 0.41 (0.19–0.91). The odds of getting functionally impaired for non-adhered to medications were about 3.2 times compared to the adherent individuals (AOR (95% CI) = 3.21 (1.71–6.05), and patients accessed their medications through out-of-pockets prevented the impairment status by 54% than patients got their medications by health insurance (AOR (95% CI) = 0.46 (0.26–0.81). Participants who had follow-up at Tibebegion Comprehensive Specialized Hospital were 3.4 times more functionally impaired than those who had follow-up at Felegehiwot and Gondar Comprehensive Specialized Hospitals (AOR(95% CI) = 3.382(1.592–7.184) (Table 4).

**Table 4:** Bivariate and multivariate analysis of variables related to functional outcome among bipolar disorder patients in Northwest Ethiopia from April to June 2021 (*N* = 411)

Variable	Functional impairment status		COR (95%CI)	*p*-value	AOR (95%CI)	*p*-value
	No	Yes				
Marital status						
Single	81	84	1	< 0.001*	1	
Married	145	46	0.338(0.217–0.0525)	**0.052**	0.383(0.171–0.857)	0.020*
Widowed	2	9	3.667(0.988–13.605)	0.682	5.045(1.002–25.412)	0.05
Divorced	17	23	1.150(0.589–2.247)		1.231(0.491–3.085)	0.657
Occupation						
Civil servant	42	18	1		1	
Private worker	18	6	0.679(0.235–1.960)	0.474	0.887(0.235–3.347)	0.86
Farmer	41	22	1.325(0.633–2.772)	0.455	1.201(0.443–3.256)	0.72
Housewife	71	33	0.979(0.495–1.933)	0.95	1.033(0.399–2.676)	0.947
Unemployed	32	55	4.451(2.219–8.929)	< 0.001**	3.911(1.457–10.499)	0.007*
Student	46	27	1.427(0.700–2.909)	0.328	0.762(0.259–2.245)	0.622
Study area						
Gondar	118	62	1	0.709	1	0.98
Felegehiwot	104	61	1.086(0.703–1.680)	< 0.001**	2.823(1.493–5.334)	0.002*
Tibebegion	25	41	2.986(1.691–5.274)		3.382(1.592–7.184)	
BMI						
Normal	190	124	1	0.695	1	0.322
Underweight	29	20	1.126(0.622–2.041)	0.386	1.488(0.678–3.266)	0.609
Overweight	26	14	0.742(0.377–1.1458)	0.040*	0.776(0.294–2.049)	0.028*
Obese	3	5	4.096(1.066–15.735)		6.491(1.219–34.575)	
Type of BD						
BD-I	195	117	1		1	0.994
BD-I with psychotic feature	42	38	1.405(0.864–2.286)	**0.171**	0.997(0.484–2.054)	0.078
BD-II	9	10	2.646(1.111–6.302)	0.028*	3.303(0.874–12.481)	
Current mood status						
Euthymic phase	170	46	1		1	
Manic phase	63	90	5.644(3.579–8.900)	< 0.001**	5.153(2.839–9.352)	< 0.001**
Depressive phase	15	27	6.453(3.265–12.752)	< 0.001**	7.794(3.313–18.338)	< 0.001**
Treatment duration						
≤ 1 year	51	53	1		1	0.051
1–5 year	130	63	0.482(0.298–0.779)	0.003*	0.500(0.249–1.004)	0.067
5–10 year	52	32	0.596(0.337–1.054)	**0.075**	0.361(0.361–1.863)	0.384
> 10 year	16	14	0.903(0.406–2.009)	0.803	0.588(0.178–1.942)	
No of relapse per year						
No relapse	164	70	1	< 0.001**	1	0.006*
Once relapse	75	78	2.494(1.640–3.792)	< 0.001**	2.235(1.254–3.984)	< 0.001**
≥ two relapses	9	15	5.067(2.186–11.744)		8.303(2.725–25.295)	
Suicidal ideation						
Yes	25	49	1	< 0.001**	1	0.008*
No	227	110	0.260(0.156–0.436)		0.415(0.216–0.795)	
Suicidal attempt						
Yes	6	19	1	< 0.001**	1	0.99
No	240	146	0.206(0.090–0.473)		1.009(0.271–3.751)	
Lifetime substance use						
Yes	119	91	1	0.084	1	0.226
No	131	70	0.709(0.479–1.048)		0.669(0.349–1.283)	
3-month substance use						
Yes	20	31	1	0.001**	1	0.028*
No	228	132	0.363(0.204–0.645)		0.413(0.188–0.908)	
No of hospitalization						
	120	63	1	**0.061**	1	1
No	86	67	1.521(0.981–2.358)	**0.071**	1.513(0.810–2.825)	0.194
Once	32	25	1.722(0.954–3.110)	**0.152**	1.502(0.604–3.735)	0.381
Twice	9	8	1.968(0.779–4.976)		0.951(0.237–3.816)	0.944
≥ three						
Adherence						
Adherent	31	60	1		1	
Non-adherent	218	102	4.496(2.763–7.317)	< 0.001**	3.212(1.706–6.048)	< 0.001**
Medication acquisition						
Health insurance	104	97	1	0.001**	1	0.007*
Out of pocket	139	71	0.515(0.347–0.764)		0.457(0.259–0.807)	
Medication discontinuation						
Yes	102	82	1	0.016*	1	1
No	146	81	0.618(0.417–0.915)		0.695(0.373–1.294	0.251
Current regimen types						
MS plus AP	160	120	1		1	1
MS plus AD	6	6	1.301(0.410–4.132)	0.656	0.877(0.147–5.222)	0.885
MS alone	20	5	0.434(0.179–1.053)	**0.065**	6.324(0.270–148.401	0.252
AP alone	45	18	0.488(0.270–0.880)	**0.017**	5.643(0.258–123.642	0.272
AP plus MD	6	3	0.557(0.141–2.200)	0.404	0.924(0.162–5.285)	0.929
MS plus AP plus AD	12	10	1.301(0.565–2.995)	0.537	1.484(0.418–188.275	0.542

*Indicates *p* < 0.05 and **indicates *p* < 0.001

## Discussion

In the present study about 40% of the study participants were functionally impaired, among the specific domains the cognitive domain was the most affected, and leisure time was the least impaired domain. Being unemployed, married, obese, having a manic and depressive mood, presence of relapse, suicidal ideation, current substance use, non-adherence, and accessing their medications out of pocket were independent predictors of functional impairments. The current study shows that the overall median (IQR) functional impairment levels of the participants was 6 (0–22).

In this study, about 40% with 95%CI of (34.7–44.2) of the bipolar patients were functionally impaired. This is similar to reports found in the USA 38% [8], and in Barcelona at different times 43% [23] and 44% [24]. In contrast, the impairment levels in our study were lower than the findings in the UK 68% [25], brazil 60% [26], and in Spain 47% [27]. Compared with findings in UK, Brazil, and Spain, the impairment level in our study had better status. The possible explanation for this discrepancy might be the difference in adherence levels. In our study the adherence level was 77.9%, however, in the UK 20–60% [28] which is relatively lower than the current findings. Furthermore, it might be due to different mood statuses recorded during the study periods. The present study showed that the majority of the patients had euthymic mood status and were capable of being functionally good. On the other hand, most of the patients' mood statuses in the comparative articles were mostly in depressive and manic phases; therefore, the functional levels could be more impaired [29, 30]. As a result, depressed patients showed fewer social interactions with friends and family, less interest or desire in leisure time activities, less autonomy to maintain duties, and poorer cognitive functioning than euthymic patients [31, 32]. Moreover, another possible reason could be some of the comparative studies used interventional and controlled study designs that potentially handled likely confounders and prevent the misestimating of functional impairment levels.

The present study revealed that a higher number of individuals were functionally impaired in cognitive and occupational domains, whereas the leisure time domain was the most preserved. This is consistent with the finding reported in Brazil [24] and Spain [19], and cognitive and occupational domains are the most severely affected. A similar study from the USA [29] also showed that significant numbers of patients with BD were functionally impaired in occupational domains. The good achievement of lower impairment status in the leisure time domain might be the bipolar patients possibly have accessed the psychotherapeutic interventions on top of the usual therapies. In the leisure time domain functioning is commonly measured by daily physical activities, such as swimming, cycling, or playing and walking every day. It also measures hobbies, visiting friends, playing cards, or other games. Usually, everyone could perform these activities either knowingly or unknowingly which makes leisure time function well [30].

On the other hand, the respondents’ impairment levels were increased in cognitive and occupational domains compared with the leisure time domain. Cognitive functioning is the most difficult to achieve, as it measures concentration, memory, arithmetic abilities, problem-solving abilities, and learning activities [33]. Those measurements of cognitive functioning are not easy, and even it is difficult to determine whether cognitive functioning is achieved or not practically. Further, the clinicians might also fail to solve concentration problems for those who could not achieve cognitive functioning. Therefore, the impairment status becomes higher in the cognitive domain. Concerning the occupational domain, bipolar patients could not satisfy with the position they hold and are paid compared to their colleagues. A qualitative study done on bipolar disorder patients also supports the above finding. Up on the interview participants tended to describe the intermittently disruptive effects of BD upon work [34].

The majority of the participants had been treated with combinations of mood stabilizers and antipsychotics and most patients were on a minimum of two medications. A similar finding was obtained from a study done in the US [35], and a multi-centered study across countries [36]. However, the combined therapy results in an undesired medication burden it had better efficacy than monotherapy. Clinical trials and treatment guidelines also support the use of combination therapy for better outcomes [37, 38]. In the present study a combination of sodium valproate and risperidone took higher proportions. These medications had good pharmacological tolerability and serum monitoring profiles than lithium [39]. In contrast, in a study done in other parts of Ethiopia, Butajira [40], most patients have been treated with old-generation antipsychotics and anti-depressants. The evidence for the effectiveness of old-generation antipsychotics for the treatment of mania is, however, limited and the current guidelines advise restricting their use to short-term therapy to decrease possible side effects [37]. The possible reason for the difference may be in Butajira the medication was donated by the projects, while in our study source of medication was via out-of-pocket and health insurance. This finding implies that for better functional outcomes close follow-up of patients (close monitoring of their moods every visit) and treating them with a combination of antipsychotics and mood stabilizers having better tolerability and non-overlapping side effect profiles is useful. The use of appropriate clinical guidelines and adherence to it is important for better and safe use of drugs in bipolar patients and enhanced treatment outcome.

Regarding socio-demographic factors, the present study revealed that being married and unemployed are substantially associated with functional impairment in bipolar patients. Married individuals had better at reducing functional impairment compared with the single individual and this is consistent with previous studies [30, 41]. A married individual could have better possibilities to build social skills, and familial support and increases their functional status. Therefore, socially supported BD patients might have a better follow-up and treatment compliance, and favor for emotional and psychological components. In addition, our study also unveiled that being unemployed was significantly associated with functional impairment. A similar finding was obtained in another study [42]. Unemployment had significant consequences on the functional outcomes through its countless deficiencies in areas of social, household, and cognitive impairments. It is also a means for financial burdens and treatment plan compromisations.

In the present study, clinical factors like obesity, current mood status, number of relapses, medication adherence, and medication acquisition were important predictors of functional impairment. Obese patients were functionally impaired compared with healthy-weight patients. A similar finding was observed in another study [43]. An increase in BMI is strongly related to chronic disease states, prolonged disease duration, and poorer functional outcomes [44]. Moreover, BD patients with higher BMI had lesser improvement in disease severity, higher anxiety disorders, chronic depressive episodes, complex medical complications, worsen physical and mental health functioning, low favorable responses to treatments, and adverse course and outcomes compared with non-obese [45]. Current mood status, being depressed, and manic were significantly associated with functional impairment compared with euthymic. This finding is consistent with the findings of other studies [29, 30]. The reason might be patients with the euthymic status are stable, compliant with their managements and treatment plans, and resulting in better functional scores in all circumstances. Patients with bipolar depression may have a greater impact on a particular area of functioning such as social and daily activities, and work productivity, and leads to unemployed and days missed from work. Bipolar depression also may have an insidious onset; however, the effect on life functioning is permanent [46]. Additionally, patients who had relapses are more susceptible to functional impairment than those who did not have relapses. This finding is in agreement with other reports that found a higher rate of relapse associated with functional impairments [40]. Relapse may interfere with daily activities and treatment compliance. Besides, the relapses could induce substantial stressful situations and anxiety conditions which further make the patients believe that the disorder is incurable and they are being mismanaged.

Medication non-adherence was among the commonest factors that are significantly associated with patients' functional impairments in the present study. This finding is consistent with the finding of previous studies [47, 48]. Poor adherence to the treatment may increase relapse rates, hospital attendance, and poor medication response and community adjustment. Furthermore, being non-adherent to the given medications might cause exacerbations of social and work-related impairments and increase the prevalence of comorbidity, prolonged depression, and compromise educational levels [49, 50]. Non-adherence also causes a decrease in QOL, increases suicidal attempts, and sharply increases both personal and financial costs [51].

In this study individuals who purchase medications using out-of-pocket had better functional levels than those who were health insured. The possible reason may be the reimbursement difference; in this regard, if the medication is not available in the health institution and the individual purchase in the private pharmacies, the government reimbursed the cost of medications, only equivalent to the cost of the health institution. This may make the patients less likely to satisfy with the use of health insurance and increases impairment levels by reducing adherence to psychotropic medications, increasing rate of emergency service, increasing hospitalization, and decreasing psychiatric medication refills, supported by a study done in the US [52]. The other reason may be some observational evidence indicated that procurer patients out of pocket were less likely to discontinue their medications and even had better alternatives when the prescribed drugs are not available in the public health institutions where they are treated. Also, some medications are even costly for patients getting their medications from health insurance and be incapable to afford the usual costs. Therefore, individuals who could afford medications out of pocket have better adherence to treatment and consequently better outcomes recorded. But, this does not mean that to recommend patients not to use health insurance, rather it indicates government would make available the medications and supplies for those health-insured individuals as much as possible.

In the current study, suicidal ideation and current substance use were important psychosocial variables potentially affect the functional impairment status (Additional file 1).

In the present study, suicidal ideation was significantly associated with functional impairments. This finding is supported by studies done in Ethiopia at St, Paulo’s hospital Addis Ababa [53]. The possible reason could be suicidal ideation has undesirable consequences for self-confidence, social relationships, stigma resistance, and day-to-day activities, which may have directly related to reduced adherence, treatment failure, and increase functional impairments. Furthermore, current substance use significantly affects the functional outcome of individuals with bipolar disorder. This finding is consistent with the finding of other studies [54, 55] different research findings revealed that found that BD patients are at risk of developing drug or alcohol-related problems that could in turn contribute to more diverse and complex clinical presentation, hastened relapses, worsening of depressive features, lesser response to treatment, increase suicidal risk and increase functionally impairments [25, 56]. Regarding the study area participants who had follow-up at Tibebegion Comprehensive and Specialized Hospitals were functionally impaired than those at Felegehiwot and Gondar Comprehensive Specialized Hospitals. The possible reason might be Tibebegion Comprehensive and Specialized Hospital was established before five years, in 2018 G.C which is the youngest hospital. However, Felegehiwot and Gondar Comprehensive Specialized Hospitals were the oldest Hospitals in Ethiopia, having senior psychiatrists and experts, and well-trained staff, supported by many non-governmental organizations that make them numerous medications than Tibebegion Hospital. In addition, the majority of bipolar II participants were found in Tibebegion which is more difficult to diagnose and manage in developing nations, like Ethiopia, than bipolar I participants.

This study has several limitations. Being cross-sectional difficult to ascertain the causal nature of predictors. There are information biases, such as recall bias, since the patients did not know their age at onset, number of relapses, number of hospitalization, and social desirability bias for disclosing self-harm. It was not assessed for psychological treatments. Charts with missing data were not included in this study, and a complete case analysis was done and may not show the true functional status of the population, and may underestimate or overestimate. We only studied the factors that affect the overall functioning of bipolar patients, but factors that affect specific domains are not studied. Since there are only 19 patients with bipolar disorder II, it is difficult to generalize the finding of this study for these groups of patients, and we recommend other researchers study bipolar I disorder patients exclusively.

## Conclusion

The current study revealed that significant numbers of individuals were functionally impaired. The median (IQR) score of functional levels lies in the normally functioning domain range 6(0–22). Leisure time was the most preserved functional domain, whereas cognitive followed by occupational domains were the most impaired functional domains. Commonly, the combinations of mood stabilizers and antipsychotics were prescribed for bipolar patients. Concerning the potential predictors of the functional outcome levels, unemployment, depressed and manic phases of the mood status, obesity, number of relapses per year, suicidal ideation, current substance use, and non-adherence were significantly associated with functional impairment of the patients. However, being married and having medication out of pocket were significantly associated with good functional outcomes. Bearing in mind the heightened importance of promoting functional outcomes, a combination of mood stabilizers and antipsychotics should be selected cautiously, and the functional levels were also monitored closely at least every follow-up throughout the treatment periods for bipolar disorder patients.

## Supplementary Information

**Additional file 1:** Part I: Insight Assessment Tool For Selecting The Eligible Respondents. Part II: Socio-Demographic Characteristics Of Participant. Part III: Clinical Characteristics Of Patients With Bipolar Disorder. Part IV: Participants social Support Level. Part V: Substance Use Level. Part VI: Suicidality History. Part VII: Medication Adherence Rating Scale (Mars) Questionnaire. Part VIII: Functioning Assessment Short Test (Fast) Questionnaire.

## Data Availability

The materials and data of this study are available from the corresponding author upon request.

## Abbreviations

BD: Bipolar disorder
FAST: Functioning Assessment Short Test
FHCSH: Felegehiwot Comprehensive Specialized Hospital
MARS: Medication Adherence Rating Scale
TGCSH: Tibebegion comprehensive specialized hospital
UoGCSH: University of Gondar Comprehensive Specialized Hospital
WHO: World Health Organization
