# Supplementary material for: Assessments of functional outcomes and its determinants among bipolar disorder patients in Northwest Ethiopia comprehensive specialized hospitals: a multicenter hospital-based study
Source: Ann Gen Psychiatry. 2023 Apr 6;22:14. doi: 10.1186/s12991-023-00444-3 (PMC10077702; doi:10.1186/s12991-023-00444-3)
Supplement: Supplementary file 1 — Additional file 1: Part I: Insight Assessment Tool For Selecting The Eligible Respondents. Part II: Socio-Demographic Characteristics Of Participant. Part III: Clinical Characteristics Of Patients With Bipolar Disorder. Part IV: Participants social Support Level. Part V: Substance Use Level. Part VI: Suicidality History. Part VII: Medication Adherence Rating Scale (Mars) Questionnaire. Part VIII: Functioning Assessment Short Test (Fast) Questionnaire. [file 12991_2023_444_MOESM1_ESM.docx]

# **Annexes**

**Annex 1.1: information sheet and informed consent**

**Introduction**

Dear participant:

My name is_________________ and I am here to collect a data on behalf of Melak Erara, a post graduate student of clinical pharmacy at University of Gondar and currently conducting a research on the “Assessments of functional outcome and its determinants among bipolar disorder patients***”***on medication therapy at UoGCSH, FCSH, and TCSH, psychiatry clinic for the partial fulfillment of the requirements for the degree of masters in clinical pharmacy. The aim of the study is to assess the of functional outcomes levels and its determinants among bipolar disorder patients at UoGCSH, FCSH, and TCSH, which in turn can serve as an input in improving the treatment progress of the patients and facilitate further studies on the subject matter. Your participation in the study is based on your will. If you don’t want to be part of it, there is no any kind of pressure you are going to face and as the same time your participation will not expose you to any danger. I just want to make you sure that the confidentiality of the information collected from you will be maintained very carefully and your name will not be registered on it. Instead of your name the data will be given its own code and stored. If you feel any discomfort at any time, you will have a full right to withdraw from the study. This questionnaire might take 15-20 minutes of your time. The participants of the study were selected based on lottery method and I hope that you will not resist being a part of this research just for the sake of the benefit expected from the result. If it’s okay for you to participate, you need to understand the consent form carefully and put your sign clearly. Then after, you will proceed with answering questions raised for you by the data collector.

**Name of the principal investigator**: Melak Erara (PG student, clinical pharmacy)

**Phone number:** +251918190835

**Email address:**[erara1980@gmail.com](mailto:erara1980@gmail.com)

**Name of adviser:**

1. Mr. Mekuannint Kassa (B. pharm, MSc, Asst. Prof)
2. Mr. Simegnew Handebo (MPH, Asst. Prof)

Are you willful to take part in this study?

1. Yes

2. No

**Informed Consent Form**

Hereby, I can confirm that I understood the content of the document and the nature of the research project. I am volunteer to participate in the study and knew that I am autonomous to withdraw from the research at any time I want.

Signature of the participant __________ Date__________

Name and signature of data collector ___________ Date__________

Name and signature of supervisor ____________ Date__________

**Part i: insight assessment tool for selecting the eligible respondents**

| **1** | Do you accept that you have mental illness? | 1. Yes | 1. No |
| --- | --- | --- | --- |
| **2** | Do you think that you require treatment? | 1. Yes | 1. No |
| **3** | Do you think, you require medications to stay well? | 1. Yes | 1. No |

**Part ii: socio demographic characteristics of participant**

|  | | | |
| --- | --- | --- | --- |
| Card No | | | _________________________ |
| Study area | | | ____________________________ |
| 1. Age in year | | | ___________year |
| 1. Patient ‘s sex | | | 1. Male 2. Female |
| 1. Residency | | | 1. Urban 2. Rural |
| 1. Religion: 1. Orthodox 2. Muslim 3. Protestant 4. Others_____________ | | | |
| 1. Marital status: 1. Single. 2. Married 3. Divorced 4. Widow/widower | | | |
| 1. Educational status: 1. Unable to write and read 2. Primary education (1-8 grade) 3 .2^ndry^ education (9-12 grade) 4. College and above | | | |
| 7. occupation | 1. No work 2. Daily labor 3. Farmer 4. Housewife 5. Seville servant  6. Student 7. Private employee 8. Retire 9. Others specify____________ | | |
| 1. Number of family | | ______________ | |
| 1. Living status: | | | 1. Living with family 2. Living with friends  3. Lives alone 4. Others specify__________ |
| 1. Shelter | | | 1. Yes, I do have 2. Now I am homeless |
| 1. Body weight | | | ___________kg |
| 1. Height | | | ___________cm |
| 1. Body mass index (BMI) | | | ___________Kg/m^2^ |

**Part iii: clinical characteristics of patients with bipolar disorder**

| 1. Bipolar subtype (diagnosis) ( from patients chart):_________________________________________ | | | | | | | | | |
| --- | --- | --- | --- | --- | --- | --- | --- | --- | --- |
| 1. Current mood status (from patients chart)­­­­­­­­­­­­­­­­­­­­­­­­­­­____________________________ | | | | | | | | | |
| 1. Presence of any medical co-morbid illness | | | | | | | 1. Yes | | 1. No |
| 1. If yes to Q 16, which co morbidity? _________________________________ | | | | | | | | | |
| 1. Previous medications for both psychiatric and medical problems | | | | | | | | | |
| 1. Current medications for both psychiatric and medical problems | | | | | | | | | |
| 1. Traditional medicines | 1. Yes 2. No | | | | | | | | |
| 1. If yes for Q 20, which traditional medicine? ____________________________ | | | | | | | | | |
| 1. Age at onset of the illness in year | | ______________ | | | | | | | |
| 1. Duration of illness | | ______________ | | | | | | | |
| 1. Duration of treatment | | _____________ | | | | | | | |
| 1. Number of relapse since diagnosis | | _______________ | | | | | | | |
| 1. Number of relapse per year | | ______________ | | | | | | | |
| 1. Medication acquisition | | | 1. Health insurance | | | | | 1. Out of pocket | |
| 1. Medication Discontinuation | | | 1. Yes | | 2. No | | | | |
| 1. Number of hospitalization due to BPD: _____________________ | | | | | | | | | |
| 1. Family history of BD | | | | 1. Yes | | 1. No | | | |
| 1. Pregnancy | | | | 1. Yes | | 1. No | | | |
| 1. Breast feeding | | | | 1. Yes | | 1. No | | | |

**Part iv: participantssocial support level**

1. How many people are you so close to that you can count on them if you have great personal problems?
2. None B) 1-2 C) 3-5 D) 6 and above
3. How much interest and concern do people show in what you do?
4. Very little B) Little C) Uncertain D) Some E) A lot
5. How easy is it to get practical help from neighbors if you should need it?
6. Very difficult B) Difficult C) Possible D) Essy E) Very easy

**Part v: subtance use levle**

1. In your life time, have you used any subtance ? 1. Yes 2. No

2. If your answer is **yes** for Q1. question, then what kind of substance have you used?

1. Alchohol 2. Chat 3. Tobacco 4. Other, specify __________________

3. In the past 3 months have you used any substances? 1. Yes 2. No

4. If your answer is yes for Q3, then what kind of substance have you used?

1. Alchohol 2. Chat 3. Tobacco 4. Other, specify ______________________

**Part vi: sucidallity history**

1. Have you seriously thought about committing suicide within the last moth? 1. Yes 2. No
2. Have you attempted to commit suicide within the last month? 1. Yes 2. No

**Part vii: medication adherence rating scale (mars) questionnaire**

| S. no. | Question | Yes | No |
| --- | --- | --- | --- |
|  | Do you ever forget to take your medication? |  |  |
|  | Are you careless at times about taking your medication? |  |  |
|  | When you feel better, do you sometimes stop taking your medication? |  |  |
|  | Sometimes if you feel worse when you take the medication,  do you stop taking it? |  |  |
|  | I take my medication only when I am sick |  |  |
|  | It is unnatural for my mind and body to be controlled by medication |  |  |
|  | My thoughts are clearer on medication |  |  |
|  | By staying on medication, I can prevent getting sick. |  |  |
|  | I feel weird, like a ‘zombie’ on medication |  |  |
|  | Medication makes me feel tired and sluggish |  |  |

**Part viii: functioning assessment short test (fast) questionnaire**

| **Types of functioning test** | Types of question | No (0) | Mild (1) | Moderate  (2) | Sever (3) |
| --- | --- | --- | --- | --- | --- |
| **Autonomy** | 1. Taking responsibility for a household |  |  |  |  |
|  | 1. Living on your own |  |  |  |  |
|  | 1. Doing the shopping |  |  |  |  |
|  | 1. Taking care of yourself (physical aspects, hygiene) |  |  |  |  |
|  |  | | | | |
| **Occupational functioning** | 1. Holding down a paid job |  |  |  |  |
|  | 1. Accomplishing tasks as quickly as necessary |  |  |  |  |
|  | 1. Working in the field in which you were educated |  |  |  |  |
|  | 1. Occupational earnings |  |  |  |  |
|  | 1. Managing the expected workload |  |  |  |  |
|  |  | | | | |
| **Cognitive functioning** | 1. Ability to concentrate on a book or film |  |  |  |  |
|  | 1. Ability to make mental calculations |  |  |  |  |
|  | 1. Ability to solve a problem adequately |  |  |  |  |
|  | 1. Ability to remember newly learned names |  |  |  |  |
|  | 1. Ability to learn new information |  |  |  |  |
|  |  | | | | |
| **Financial issues** | 1. Managing your own money |  |  |  |  |
|  | 1. Spending money in a balanced way |  |  |  |  |
|  |  | | | | |
| **Interpersonal relationship** | 1. Maintaining a friendship or friendships |  |  |  |  |
|  | 1. Participating in social activities |  |  |  |  |
|  | 1. Having good relationships with people close to you |  |  |  |  |
|  | 1. Living together with your family |  |  |  |  |
|  | 1. Having satisfactory sexual relationships |  |  |  |  |
|  | 1. Being able to defend your interests |  |  |  |  |
|  |  | | | | |
| **Leisure time** | 1. Doing exercise or participating in sport |  |  |  |  |
|  | 1. Having hobbies or personal interests |  |  |  |  |

**Annex 1.2: Declaration**I, the under signed, MSc student declared that this proposal is my original work in partial fulfillment of the requirement for the master of degree in clinical pharmacy.

Name: Melak Erara
Signature: ____________________
Place of submission: School of pharmacy, College of Medicine and Health Science, University
of Gondar.
Date of submission: ________________
this proposal work will be submitted for examination with our approval as University Advisors.

**Advisors’ Name Date Signature __________________________ _________________ _____________**

- - - 1. Mr. Mekuannint Kassa (B. pharm, MSc, Asst. Prof) ___________ ____________
      2. Mr. Simegnew Handebo (MPH, Asst. Prof)______________________ ___________

**Examiners’ name** **Date** **Signature**
 **_______________________ ________________ _____________**

**Annex 1.3: Assurance of the investigator**
The undersigned student of Master of Pharmacy in Clinical Pharmacy agree to accept
responsibility for the scientific, ethical and technical conduct of the research project and for
provision of required progress reports as preterm and condition of research and publications office of the University of Gondar.
**Student’s Name** **Date** **Signature**
Melak Erara ________________________ ______________ _____________

**Approval of Advisors’**

Mr. Mekuannint Kassa (B. pharm, MSc, Asst. Prof) _______________ ____________

Mr. Simegnew Handebo (MPH, Asst. Prof) ______________ ___________ **Examiners’ Name Date Signature**
1. **______________________ ________________ _____________**2. **_______________________ ________________ _____________**
